# miR-193b regulates tumorigenesis in liposarcoma cells via PDGFR, TGFβ, and Wnt signaling

**DOI:** 10.1038/s41598-019-39560-0

**Published:** 2019-03-01

**Authors:** Ying Z. Mazzu, Yulan Hu, Yawei Shen, Thomas Tuschl, Samuel Singer

**Affiliations:** 1 Department of Surgery, Memorial Sloan Kettering Cancer Center, New York, NY USA; 2 Laboratory of RNA Molecular Biology, The Rockefeller University, New York, NY USA

## Abstract

Liposarcoma is the most common soft tissue sarcoma. Molecularly targeted therapeutics have had limited efficacy in liposarcomas, in part because of inadequate knowledge of the complex molecular alterations in these tumors. Our recent study revealed the tumor suppressive function of miR-193b in liposarcoma. Considering the biological and clinical heterogeneity of liposarcoma, here, we confirmed the under-expression of miR-193b in additional patient liposarcoma samples and cell lines. Based on STRING analysis of protein-protein interactions among the reported putative miR-193b targets, we validated three: PDGFRβ, SMAD4, and YAP1, belonging to strongly interacting pathways (focal adhesion, TGFβ, and Hippo, respectively). We show that all three are directly targeted by miR-193b in liposarcoma. Inhibition of PDGFRβ reduces liposarcoma cell viability and increases adipogenesis. Knockdown of SMAD4 promotes adipogenic differentiation. miR-193b targeting of the Hippo signaling effector YAP1 indirectly inhibits Wnt/β-catenin signaling. Both a PDGFR inhibitor (CP-673451) and a Wnt/ β-catenin inhibitor (ICG-001) had potent inhibitory effects on liposarcoma cells, suggesting their potential application in liposarcoma treatment. In summary, we demonstrate that miR-193b controls cell growth and differentiation in liposarcoma by targeting multiple key components (PDGFRβ, SMAD4, and YAP1) in several oncogenic signaling pathways.

## Introduction

Liposarcomas, arising within adipose tissue, are the most common soft tissue sarcoma, accounting for about 20% of all adult sarcomas. They are subclassified according to their histology and molecular signature into four distinct subsets: well-differentiated liposarcoma (also known as atypical lipomatous tumor); dedifferentiated liposarcoma; myxoid/round cell liposarcoma; and pleomorphic liposarcoma^1^. Well-differentiated liposarcoma (WDLS) and dedifferentiated liposarcoma (DDLS) constitute the most common biologic group of liposarcomas, and 90% of WDLS and DDLS carry amplification of chromosome 12q13-15^2^. WDLS tends not to metastasize, but can recur locally. However, if WDLS dedifferentiates into DDLS, it becomes more aggressive and acquires the potential to metastasize. WDLS and DDLS thus offer an intriguing window on molecular mechanisms driving liposarcoma progression and metastasis.

The primary management of WDLS/DDLS is surgical resection, since conventional chemotherapy has low response rates and does not extend survival^3^. Effective targeted treatment strategies are desperately needed for patients with advanced disease. Developing these specific therapies requires elucidating the molecular dysregulation underlying liposarcomagenesis. One area that could inform the development of new treatments is dysregulation of microRNAs (miRNAs), which are small non-coding RNAs that induce posttranscriptional regulation of target genes^4^. Several miRNAs have been found to have significantly altered expression in well-differentiated and dedifferentiated liposarcoma compared to normal fat tissue through deep RNA sequencing and microarray studies by our group and others^5–8^. miRNAs can function as oncogenes or tumor suppressors, depending on their target genes. Moreover, miRNAs can be used as biomarkers for tumor diagnosis, prognosis, or even as therapeutic targets^9,10^.

The functions of some miRNAs that are dysregulated in liposarcoma have been identified, while others’ contribution to tumor progression remains unknown. Underexpressed miR-143, miR-145, and miR-451 function as tumor suppressors in liposarcoma cells^5,7^, while overexpressed miR-155 and miR-26a-2 promote liposarcoma tumorigenesis^6,11^. Previously we found that miR-193b is significantly downregulated in DDLS, in part because of hypermethylation of its promoter region, and that miR-193b functions as a tumor suppressor by targeting multiple key oncogenes^12^. In the current study, we report three new signaling pathways (PDGFR, TGFβ, and Wnt) targeted by miR-193b in liposarcoma, which could contribute to miR-193b’s functions as a tumor suppressor by inhibiting proliferation and promoting adipogenic differentiation in WDLS cells and adipose-derived stem cells (ASCs).

## Results

### miR-193b is underexpressed in liposarcoma tissues and cell lines

**Figure 1:**
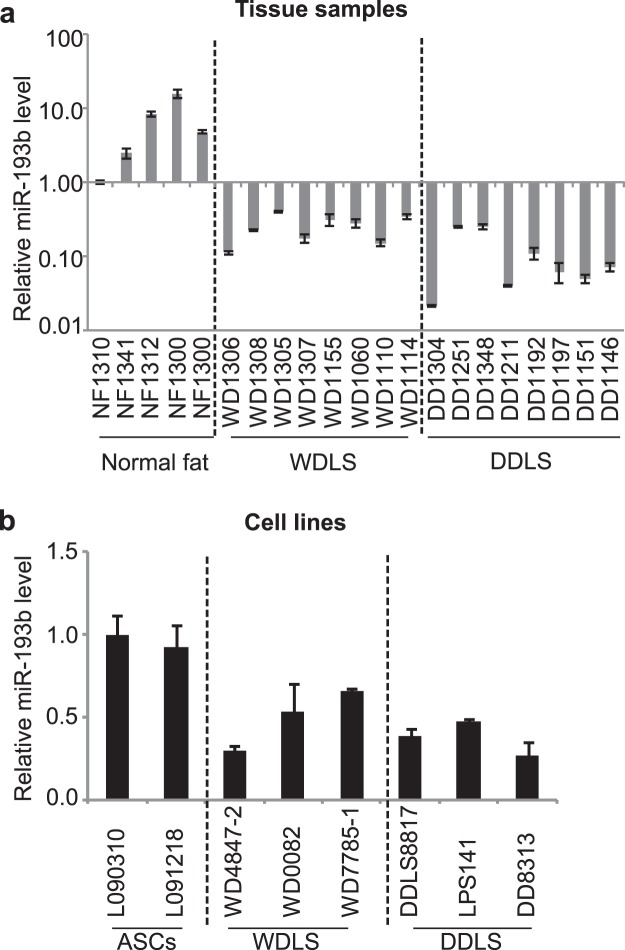
miR-193b is underexpressed in liposarcoma tissue and cell lines. (**a**) miR-193b expression in normal fat, WDLS, and DDLS tissues. (**b**) miR-193b expression in ASCs, WDLS, and DDLS cell lines. Expression was normalized relative to expression of U6 small RNA, and normalized values were then expressed relative to the level of miR-193b in the NF-1310 sample for tissues, and to that in the L090310 ASC line for cells. Values represent the mean ± S.E. of three independent experiments.

We have previously shown by deep RNA sequencing that miR-193b is underexpressed in DDLS and a subset of WDLS tumors^12^. RT-PCR confirmed lower miR-193b expression in patient tumor samples (Fig. 1a; WDLS samples with low expression of this miRNA were selected for analysis). In WDLS and DDLS cell lines, miR-193b levels were similarly decreased compared with the normal cell control, adipose-derived stem cells (ASCs; Fig. 1b).

### miR-193b overexpression inhibits growth of DDLS and WDLS cells via key targets that regulate crosstalk of oncogenic pathways

**Figure 2:**
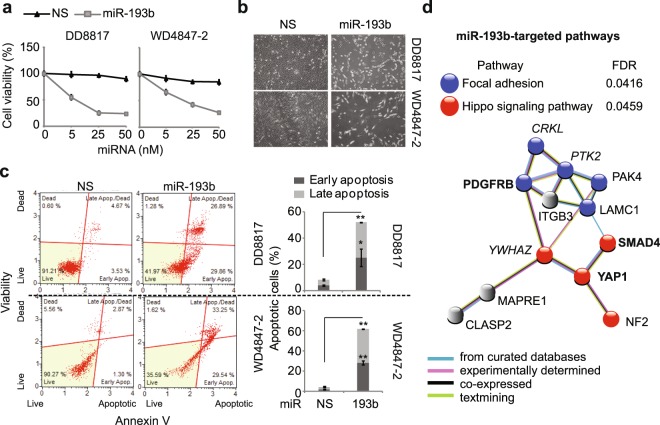
miR-193b functions as a tumor suppressor in liposarcoma cells. (**a**) Cell viability on day 5 after transfection of miR-193b or a nonspecific control miRNA (NS) into liposarcoma cells at various doses. (**b**) Phase-contrast photographs of liposarcoma cells transfected with NS or miR-193b on day 5 (10x magnification). (**c**) Apoptosis of miR-193b-treated liposarcoma cells detected by annexin V assays. Values represent the mean ± S.E. of three independent experiments. **p < 0.01 compared with cells treated with nonspecific miRNA. (**d**) STRING protein-protein interaction network analysis of putative miR-193b targets. Network nodes represent proteins. Same-colored target genes are enriched in the same pathway. Edges represent protein-protein associations, colored according to association types. Putative targets in italics are previously validated; those in bold were further investigated in this study.

As reported previously^12^, overexpression of miR-193b significantly inhibited DD8817 and WD4847-2 cell growth in a dose-dependent manner (Fig. 2a). At 3 days post-transfection, 25 nM miR-193b inhibited cell viability by 50%. miR-193b-induced apoptosis was observed (Fig. 2b) and measured by the Annexin V assay. Compared to control miRNA, miR-193b transfection increased apoptosis from 4–8% to about 60% in DDLS and WDLS cells (Fig. 2c). These results confirm that miR-193b functions as a tumor suppressor in liposarcoma cells.

In our previous study, we detected transcriptomic and proteomic changes in miRNA-treated liposarcoma cells and identified 50 potential miR-193b targets using multiple miRNA target prediction tools^12^. To further understand miR-193b target networks, we applied the functional protein-protein interaction analysis method STRING, which revealed interactions among multiple signaling pathways (Fig. S1). Among them, focal adhesion and Hippo signaling are the top two, with the lowest false discovery rate values. Our prior study identified members of the focal adhesion pathway, FAK (PTK2) and CRKL, as direct targets of miR-193b^12^; STRING analysis indicated that miR-193b targets both of those as well as PDGFRβ to repress the focal adhesion pathway (Fig. 2d). STRING also implicated SMAD4 and Yes-associated protein (YAP)-1, members of the Hippo pathway, as new targets of miR-193b, in addition to the previously identified YWHAZ^13^. These results suggest that these three novel miR-193b targets may interact with previously identified miR-193b targets to modulate multiple oncogenic signaling pathways in liposarcoma.

### PDGFRβ is a direct target of miR-193b and inhibition of its activity attenuates differentiation and proliferation of liposarcoma cells

**Figure 3:**
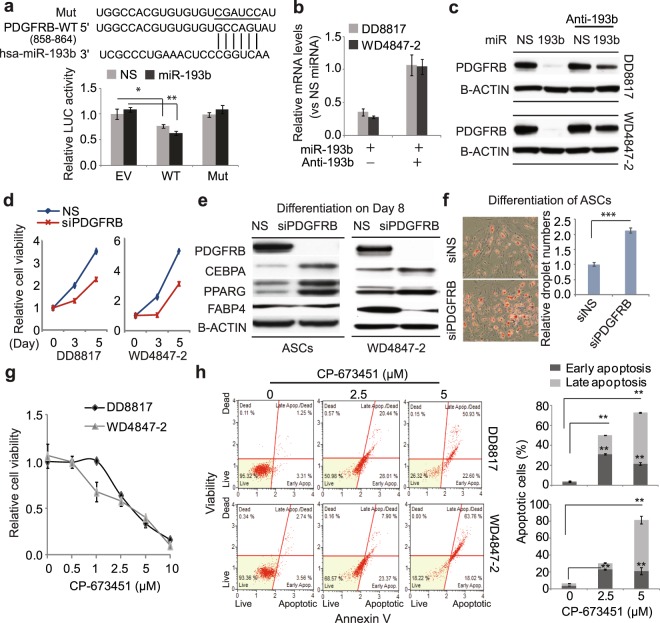
PDGFRβ is the direct target of miR-193b and functions as an oncogene in liposarcoma cells. (**a**) Direct binding of miR-193b to the PDGFRβ 3′UTR was tested by luciferase reporter assays. The reporters contain either the luciferase gene alone (EV) or a PDGFRβ 3′UTR fragment with wild type (WT) or mutant (Mut) miR-193b seed sites. Luciferase activity was measured after 48 h co-transfection of miRNAs and reporters. Values were normalized to NS miRNA plus EV. (**b**,**c**) PDGFRβ mRNA (**b**) and protein levels (**c**) in liposarcoma cells treated with miR-193b with or without antimiRNA. (**d**) Effects of PDGFRβ knockdown on liposarcoma cell viability. Values were normalized to day 0. (**e**) Effects of PDGFRβ siRNA on adipogenic differentiation in ASCs and WD4847-2 cells. Adipogenic differentiation was induced 3 days after siRNA transfection, and protein levels were analyzed on day 8 of differentiation. (**f**) Lipid droplet formation (Oil red O staining) in siRNA-treated ASCs on day 10 of adipogenic differentiation. (**g**) Effects of PDGFRβ inhibitor CP-673451 on liposarcoma cell viability. Increasing doses of CP-673451 were applied to both DD8817 and WD4847-2 cells, and cell viability was measured on day 3 of drug treatment. (**h**) Apoptosis in CP-673451-treated liposarcoma cells detected by annexin V assays. Values represent the mean ± S.E. of three independent experiments. *p < 0.05; **p < 0.01; ***p < 0.001 compared with control groups treated with nonspecific siRNA or vehicle.

PDGFR signaling plays a crucial role in cancer development and progression^14^, so regulation of PDGFRβ by miR-193b could contribute to liposarcoma progression. To test this hypothesis, we overexpressed miR-193b in liposarcoma cells. Overexpression of miR-193b inhibited wild-type PDGFRβ-3′UTR reporter (WT) activity by 50% more than the reporter lacking the 3′UTR region (Fig. 3a). Mutation of seed sites in the 3′UTR reporter (Mut) completely blocked the repression induced by miR-193b (Fig. 3a), suggesting that miR-193b regulates PDGFRβ through its seed sites in the 3′UTR. Consistently, both mRNA and protein levels of PDGFRβ were repressed by miR-193b overexpression, while addition of anti-miRNA for miR-193b attenuated this regulation (Fig. 3b,c). These results demonstrate that miR-193b directly regulates PDGFRβ expression in WDLS/DDLS cells.

Besides suppressing tumor growth, miR-193b has been reported to promote brown fat differentiation in mouse cells^13^. Confirming our previous findings that miR-193b induces adipogenic differentiation in human adipose tissue-derived stem cells (ASCs)^12^, overexpression of miR-193b significantly increased both mRNA and protein levels of the adipogenic markers CEBPA, PPARG, and FABP4 (Fig. S2a). These markers were also induced by culture in differentiation medium (Fig. S2b). To assess PDGFRβ’s role in miR-193b-mediated growth inhibition and adipogenic differentiation, we used siRNAs to knock down endogenous PDGFRβ in WDLS and DDLS cells. PDGFRβ knockdown significantly inhibited cell viability (by 30–40% at day 5) in both cell types (Fig. 3d). In addition, knockdown of PDGFRβ promoted adipogenic differentiation in both WDLS and ASCs, evident as increased CEBPA and PPARG protein expression and lipid droplet formation (Fig. 3e,f). Interestingly, PDGFRβ siRNA reduced levels of the adipogenic marker FABP4 in WDLS, but had no effects on FABP4 in ASCs (Fig. 3e), indicating that PDGFRβ may differentially regulate the differentiation of tumor cells versus normal cells. These data together suggest that miR-193b targets PDGFRβ to regulate cell proliferation and differentiation in WDLS/DDLS and ASCs.

We further tested whether blocking PDGFR signaling using small molecules could affect WDLS/DDLS growth. The PDGFRβ inhibitor CP-673451 reduced viability of both DDLS and WDLS cells in a dose-dependent manner (Fig. 3g). To determine whether this decrease in viability involved apoptosis, we used propidium iodide and Annexin V staining. CP-673451 treatment (2.5 μM) caused 50% of DDLS and 30% of WDLS cells to undergo apoptosis, and 5 μM CP-673451 induced about 80% apoptosis in both cell types after 3 days of treatment (Fig. 3h). These results indicate that PDGFR signaling is essential for WDLS and DDLS cell survival.

### miR-193b targets SMAD4 to regulate adipogenic differentiation

**Figure 4:**
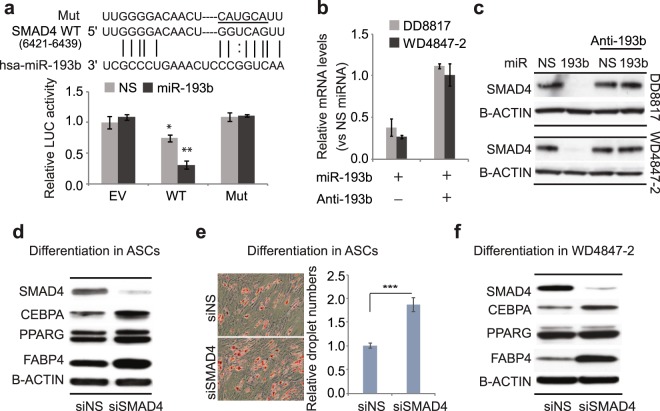
miR-193b directly blocks SMAD4 expression to inhibit adipogenic differentiation. (**a**) Luciferase reporter-based assay of direct binding of miR-193b to seed sites on the SMAD4 3′UTR. The reporters contain either the luciferase gene alone (EV) or a SMAD4 3′UTR fragment containing the wild-type (WT) or mutant (Mut) miR-193b seed site. Luciferase activity was measured after 48 h co-transfection of miRNAs and reporters, and values were normalized to NS miRNA plus EV. (**b**,**c**) SMAD4 mRNA (**b**) and protein levels (**c**) in liposarcoma cells treated with miR-193b with or without antimiRNA. (**d**) Effects of SMAD4 knockdown on protein levels of adipogenic markers in differentiated ASCs. (**e**) Formation of lipid droplets (Oil red O staining) during adipogenic differentiation of ASCs on day 10. (**f**) Effects of SMAD4 knockdown on protein levels of adipogenic markers in WD4847-2 cells. Gene expression levels were normalized to the levels of the NS group. Values represent the mean ± S.E. of three independent experiments. *p < 0.05; **p < 0.01, ***p < 0.001 vs. control groups.

SMAD4 is a central regulator of TGFβ signaling, and our STRING analysis indicated that it also interacts with YAP1 to regulate Hippo signaling. To determine whether miR-193b directly targets SMAD4, we used luciferase reporters. Addition of the wild-type SMAD4 sequence (WT) to the reporter resulted in a 25% reduction in luciferase activity, indicating that endogenous miR-193b recognizes the SMAD4 3′UTR (Fig. 4a). Compared to non-specific miRNA, transfection of miR-193b inhibited the luciferase activity of the reporter containing the WT SMAD4 3′UTR by 70% (Fig. 4a), confirming direct action of miR-193b on SMAD4 mRNA. Luciferase activity of the reporter containing a mutated version of the seed site for miR-193b was similar to that of the control, luciferase-only reporter, indicating specificity of miR-193b binding. The transfected miR-193b repressed mRNA and protein expression of SMAD4, while addition of anti-miR completely blocked the effects (Fig. 4b,c). Together, these results suggest that SMAD4 is a direct target of miR-193b in WDLS/DDLS cells.

Since miR-193b significantly reduces WDLS/DDLS cell viability, we tested whether inhibition of SMAD4 expression has similar effects. Knockdown of SMAD4 using SMARTpool SMAD4 siRNA had no effect on the viability of DDLS and WDLS cells compared with control siRNA (Fig. S3).

Knockdown of SMAD4 promoted adipogenic differentiation in ASCs as indicated by increased adipogenic marker expression and lipid droplet formation (Fig. 4d,e). This result indicates that, although inhibition of SMAD4 does not affect tumor cell viability (Fig. S3), miR-193b may regulate tumor differentiation by targeting SMAD4. To test this hypothesis, we knocked down SMAD4 before inducing differentiation in WDLS cells. Knockdown of SMAD4 indeed upregulated adipogenic markers in WDLS cells (Fig. 4f). These results indicate that miR-193b promotes adipogenesis of ASCs and regulates differentiation in WDLS/DDLS by directly targeting SMAD4.

### miR-193b directly inhibits YAP1 to attenuate the activity of Wnt/β-catenin signaling

**Figure 5:**
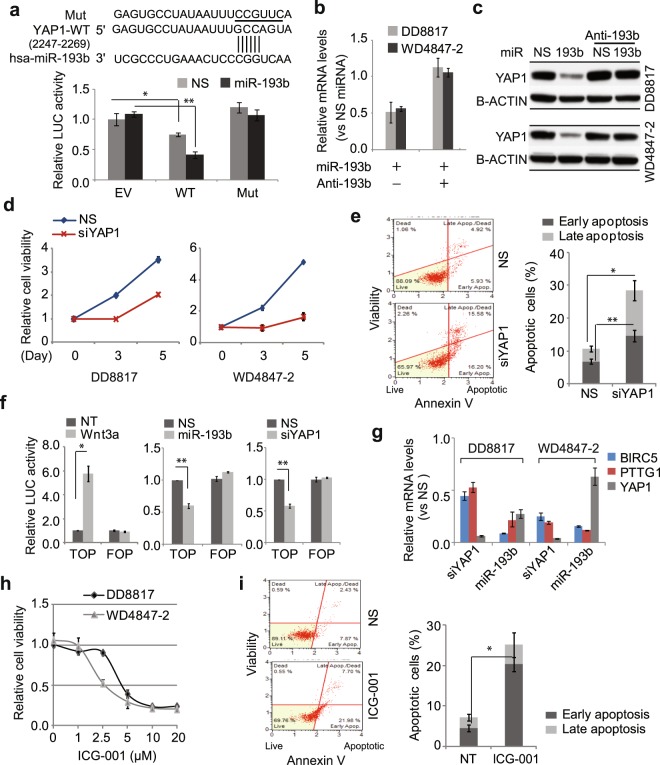
miR-193b indirectly inhibits Wnt/β-catenin signaling by targeting YAP1. (**a**) Luciferase reporter-based assay of direct binding of miR-193b to the YAP1 3′UTR. The reporters YAP1 3′UTR fragment containing the wild-type (WT) or mutant (Mut) miR-193b seed site. Reporters contain either the luciferase gene alone (no seed site) or a YAP1 3′UTR fragment containing the wild-type (WT) or mutant (Mut) miR-193b seed sites. Luciferase activity was measured after 48 h co-transfection of miRNAs and reporters, and values were normalized to NS miRNA plus EV. (**b**,**c**) YAP1 mRNA (**b**) and protein (**c**) were measured in cells treated with miR-193b or miR/antimiR-193b. (**d**) Effects of YAP1 knockdown on liposarcoma cell viability, expressed relative to that on day 0. (**e**) Apoptosis in YAP1 siRNA-treated WD4847-2 cells. (**f**) Effects of miR-193b and YAP1 siRNA on β-catenin/TCF-dependent transcription activity. As a positive control, cells were treated with Wnt3a (100 ng/mL) for 24 h (left panel). Luciferase activity is expressed relative to that in untreated (NT) or nonspecific miRNA or siRNA (NS)-treated cells. (**g**) Effects of miR-193b or YAP1 siRNA on Wnt/β-catenin target gene expression, as detected by qRT-PCR. Relative mRNA levels were obtained by comparing to those in nonspecific miRNA- or siRNA-treated cells. (**h**) Effects of Wnt/β-catenin inhibitor ICG-001 on liposarcoma cell viability. Increasing doses of ICG-001 were applied to both DD8817 and WD4847-2 cells, and cell viability was measured on day 3 of drug treatment and expressed relative to that in vehicle-treated cells. (**i**) Apoptosis in ICG-001 (2.5 µM)-treated WD4847-2 cells detected by annexin V assay on day 2. Values represent the mean ± S.E. of three independent experiments. *p < 0.05; **p < 0.01 compared with vehicle or nonspecific miRNA or siRNA control.

Overexpression or overactivation of YAP1 has been associated with tumor progression and worse survival in multiple cancers^15–19^. Amplification of *YAP1* has been observed in 16% of DDLS samples, and amplification is correlated with overexpression^20^. YAP and its coactivator (TAZ) are overactivated in several sarcoma types^21^. miRNA-mediated post-transcriptional regulation of YAP1 may thus contribute to liposarcoma progression. To determine whether YAP1 is a direct target of miR-193b, we first employed YAP1 3′UTR activity assays. Endogenous miR-193b reduced YAP1-WT reporter activity by about 20%. Transfected miR-193b inhibited 60% of YAP1-WT activity, but had no effect on YAP1-Mut reporter activity, indicating that miR-193b regulates YAP1 mRNA through its 3′UTR (Fig. 5a). Furthermore, transfected miR-193b downregulated YAP1 expression at both the mRNA and protein levels, which was blocked by addition of antimiR-193b (Fig. 5b,c). These results demonstrate that miR-193b directly targets YAP1 in WDLS/DDLS cells.

To evaluate YAP1 function, we knocked down its expression using siRNA in DDLS and WDLS cells. YAP1 knockdown significantly reduced the viability of both DDLS and WDLS cells (Fig. 5d). YAP1 siRNA also caused apoptosis in about 30% of WDLS cells (Fig. 5e), but only induced modest cell cycle arrest at G0/G1 in DDLS cells (Fig. S4). YAP1 forms a complex with β-catenin to activate downstream gene expression^22^. To examine whether miR-193b regulates Wnt/β-catenin signaling in WDLS/DDLS cells, we employed the TOP-flash/FOP-flash system, in which the TOP-flash reporter indicates Wnt activity, while the FOP-flash reporter (lacking the β-catenin binding site) serves as a negative control. Recombinant human Wnt3a significantly increased TOP-flash activity but had no effects on FOP-flash (Fig. 5f). Both overexpression of miR-193b and knockdown of YAP1 reduced TOP-flash activity by 50% but did not affect that of FOP-flash (Fig. 5f).

To confirm the regulation of Wnt/β-catenin signaling by miR-193b and YAP1, we further analyzed β-catenin target gene expression in miR-193b and YAP1 siRNA-treated DDLS and WDLS cells. YAP1 mRNA levels were significantly reduced by either miR-193b or YAP1 siRNA in both cell lines (Fig. 5g). Accordingly, the β-catenin target genes BIRC5 and PTTG1 were downregulated more than 50% in miR-193b or YAP1 siRNA-treated DDLS and WDLS cells (Fig. 5g), suggesting that miR-193b regulates Wnt/β-catenin signaling by inhibiting the Hippo regulator YAP1 in WDLS/DDLS cells.

To evaluate whether inhibition of Wnt/β-catenin signaling affects WDLS/DDLS cell viability, we applied the Wnt/β-catenin inhibitor ICG-001 to both DDLS and WDLS cells. ICG-001 (2.5 µM) reduced cell viability to 50% (Fig. 5h) and caused apoptosis in 27% of WDLS cells (Fig. 5i). A higher dose (5 µM) of ICG-001 reduced viability by 75% in both DDLS and WDLS cells (Fig. 5h). The fact that knockdown of YAP1 induced apoptosis in WDLS but not DDLS cells (Fig. 5e), but ICG-001 induced apoptosis in both WDLS cells and DDLS cells (Figs 5i and S5) suggests that YAP1 may affect Wnt/β-catenin signaling differently in WDLS vs. DDLS cells due to their different biological backgrounds.

Considering the heterogeneity of liposarcoma, we evaluated the function of miR-193b in additional liposarcoma cells (LPS141, RDD8107, WD7785-1). As shown in our previous study, endogenous miR-193b expression in these three cell lines is higher than that in DD8817 and WD4847-2, but much lower than that in ASCs^12^. Consistent with the results in DD8817 and WD4847-2 cells, miR-193b significantly inhibited cell growth in a dose-dependent manner (Fig. S6a) and induced approximately 50% reductions in target gene expression (PDGFRΒ, SMAD4, YAP1) (Fig. S6b). These results suggest that miR-193b functions similarly across multiple liposarcoma cell lines.

## Discussion

In this study, we demonstrated that miR-193b regulates multiple signaling pathways, including PDGFR, TGFβ, and Wnt, by targeting the key regulators PDGFRβ, SMAD4, and YAP1 to reduce cell viability and promote adipogenic differentiation in WDLS and ASCs. Based on these results, we showed that inhibitors of PDGFR and Wnt/β-catenin signaling significantly reduce WDLS/DDLS cell viability and induce apoptosis.

Dysregulation of miR-193b is frequently observed in cancers. As a tumor suppressor, miR-193b has been reported to target cyclin D1 to regulate the cell cycle^23^, or directly inhibit oncogenes such as Mcl-1, c-KIT, MYB, and KRAS to attenuate tumorigenesis^24–27^. Although proteins whose expression is regulated directly by miR-193b have been identified using quantitative iTRAQ analysis in breast cancer cells, there are still many potential targets that have not been validated or identified in other cancers. We recently reported that miR-193b functions as a tumor suppressor in liposarcoma by targeting the FAK-SRC-CRKL axis^12^, and here, we identify three novel targets of miR-193b (PDGFRβ, SMAD4, and YAP1), which respectively play key roles in PDGFR, TGFβ, and Hippo/Wnt signaling.

Overactivity of PDGFR signaling is observed in sarcoma, and may drive tumor growth^28,29^. Consistent with that function, we found that inhibition of PDGFRβ expression reduces DDLS and WDLS cell viability. We also observed that, similar to SMAD4, knockdown of PDGFRβ promotes adipogenesis in both ASCs and WDLS cells, which is consistent with the function of PDGFRβ in adipogenic commitment of MSCs. Thus, unlike TGFβ/SMAD4 signaling, PDGFR signaling may regulate tumorigenesis by controlling both cell viability and differentiation in WDLS/DDLS. The silencing of miR-193b that occurs in DDLS or a subset of WDLS could thus break multiple control mechanisms to keep liposarcoma cells from differentiating, and thereafter drive liposarcoma cell proliferation to promote tumor progression.

SMAD4 is a central mediator of TGFβ and bone morphogenetic protein (BMP) signaling pathways, which regulate differentiation, among other physiological processes. SMAD4 acts as a tumor suppressor in carcinomas of the pancreas and GI tract, where deletions or inactivating mutations in the SMAD4 gene have been reported^30–32^. However, SMAD4 was recently reported to exert a tumor-promoting role in hepatocellular carcinoma^33^. In WDLS/DDLS cells, knockdown of SMAD4 did not affect cell viability, but did promote adipogenic differentiation in ASCs and WDLS cells. This finding is consistent with recent studies showing that SMAD4 is essential for mouse adipogenesis^34^. Instead of affecting tumor growth, increased levels of SMAD4, as would result from the under-expression of miR-193b in DDLS, may contribute to liposarcoma progression and deregulation of differentiation in WDLS/DDLS.

Besides directly targeting key upstream regulators of the above signaling pathways, miR-193b also indirectly inhibits Wnt/β-catenin signaling by repressing the Hippo effector YAP1. YAP1 is reported to be essential for Wnt/β-catenin signaling activation in some cancer types^22,35,36^. Here, we demonstrated that miR-193b inhibits Wnt/β-catenin signaling activity by targeting YAP1 in WDLS/DDLS cells.

STRING analysis revealed protein-protein interactions among the identified miR-193b targets (Fig. 1d), suggesting crosstalk of the related signaling pathways, as has been suggested by other studies. PDGFR is known to activate FAK to promote migration^37^, and signaling downstream of both kinases converges on MEK/ERK to support proliferation^38^. Similarly, cooperation of Hippo (YAP1) and TGFβ (SMADs) signaling has been found to promote carcinogenesis^39,40^. Not only does YAP1 regulate Wnt signaling, SMAD4 also controls proteasomal degradation of β-catenin^41^. Thus, miR-193b may dampen signaling via multiple oncogenic pathways, suggesting that combinations of inhibitors of FAK, PDGFR, TGFβ, and/or YAP1 may be more effective than single drugs. Given the challenge of identifying combinations that are safe and effective, administration of miR-193b itself (or a pharmacologically optimized version thereof) may represent a promising alternative, as it would function similarly to a combination of inhibitors.

We showed that the specific PDGFRβ inhibitor CP-673451 reduces viability and induces apoptosis in the tested WDLS/DDLS cells. However, the limited activity of similar agents in the clinic suggests that such drugs may only be effective for a subset of patients with these liposarcomas^42–45^. Responses of DDLS patients to pazopanib (which inhibits PDGFR, c-KIT, FGFR, and VEGFR) were better than those in myxoid liposarcoma, suggesting that the contribution of PDGFR signaling to DDLS progression is clinically relevant^44^. As we selected cell lines with low miR-193b expression, PDGFR or multi-kinase inhibitors may be especially effective in this subset of WDLS/DDLS. Studies in patient-derived orthotopic xenograft (PDX) models would provide further evidence in support of this idea; this approach has been used to show that PDGFRα-amplified pleomorphic liposarcoma is especially sensitive to pazopanib^46^.

In summary, miR-193b functions as a tumor suppressor in WDLS/DDLS. Loss of miR-193b expression appears to attenuate inhibitory control of multiple oncogenic signaling pathways (e.g. PDGFR, TGF, and Wnt) through the key regulators PDGFR, SMAD4, and YAP1. For the first time, oncogenic functions of these regulators are confirmed in liposarcoma cells. Restoring miR-193b activity or inhibition of PDGFRβ or Wnt/β-catenin signaling could represent a new therapeutic strategy in liposarcoma.

## Methods

### Patient samples

The study was approved by the MSKCC Institutional Review Board, and all participants gave written informed consent that their tissue samples could be used for this research, in accordance with HHS guidelines and the Declaration of Helsinki. Tumor and normal adipose tissue samples (all retroperitoneal) obtained during surgical resection were snap-frozen in liquid nitrogen and embedded in cryomolds. According to MSKCC’s two-grade system, all WDLS tumors were low-grade and all DDLS tumors were high-grade. All tumors were primary except for DD1348, which was a local recurrence.

### Cell culture

Liposarcoma cell lines were established from tissue samples obtained from consenting patients: DD8817, LPS141, RDD8107, and DD8313 from DDLS samples; WD4847-2, WD8200, and WD7785-1 from WDLS samples. Amplification of 12q was confirmed by array comparative genomic hybridization. Primary human adipose tissue-derived stromal/stem cells (ASCs) were isolated from subcutaneous fat tissue samples from consenting patients as previously described^47^. The cell lines were maintained as described^5^.

### Antibodies and reagents

The sources of antibodies were as follows: PDGFRΒ (polyclonal, catalog number sc-432), vinculin (monoclonal, sc-73614), cyclin D1 (monoclonal, sc-8396), and cyclin A (polyclonal, sc-751) from Santa Cruz Biotechnology; SMAD4 (monoclonal, #38454, raised against peptide corresponding to residues surrounding D165), YAP1 (polyclonal, #4912), C/EBPα (polyclonal, #2295), FABP4 (polyclonal, #2120), PPARγ (#2430, raised against peptide corresponding to residues surrounding D69), and p-RB (Ser780) (polyclonal, #9307, raised against phosphopeptide corresponding to residues surrounding S780) from Cell Signaling Technology; and anti-RB (polyclonal, 09–100, raised against linear protein corresponding to human RB-like protein 2 at and around the C-terminus) from EMD Millipore. miRNAs (nonspecific miRNA, miR-193b) and antimiRNAs (nonspecific antimiRNA, anti-miR-193b) were purchased from Ambion. Smartpool siRNAs for SMAD4, PDGFRΒ and YAP1 were purchased from Dharmacon. CP-673451 and ICG-001 were purchased from Selleck Chemicals.

### Plasmid construction

The pmirGLO plasmid (Promega, Madison, WI) was used for PDGFRβ, SMAD4, and YAP1 3′UTR reporters. Synthetic ~100-bp oligonucleotides representing regions of the SMAD4, PDGFRβ and YAP1 3′ UTRs containing miR-193b seed motifs were inserted into the Pmel/Xbal site of pmirGLO. Mutations of the same seed sequences in reporters were also generated. The oligonucleotide sequences are shown in Table S1. The TOPflash/FOPflash reporter plasmid system was applied for monitoring β-catenin-driven Wnt transcriptional activity. The TOPFlash reporter containing TCF binding sites is activated by β-catenin, and the FOPFlash with mutated TCF binding sites serves as a negative control. Both TOPflash and FOPflash were purchased from Upstate Biotechnology (NY).

### Transient transfection and 3′UTR luciferase reporter assays

MicroRNAs and corresponding inhibitors (Ambion) were transfected into 50% confluent cells with Oligofectamine at a concentration of 50 nM (Invitrogen). Protein and mRNA were collected at 72 h after transfection.

For 3′UTR luciferase reporter assays, miRNAs or siRNAs were co-transfected with 200 ng of reporters using Lipofectamine 2000 (Invitrogen). At 48 h post-transfection, cells were collected for luciferase assays. For TOP-/FOP-Flash assays, reporters were transfected 24 h after miRNA or siRNA and cells were collected for measurement 24 h later. phRL-null Renilla luciferase plasmid was co-transfected for normalization. The luciferase activities were detected by using the dual luciferase reporter assay system according to the instructions from Promega.

### Cell proliferation and cell cycle analysis

Cell proliferation was evaluated by using the CellTiter-Glo cell viability assay (Promega) following the manufacturer’s instructions. Briefly, 1500 cells/well were plated in 96-well plates in the presence of miRNAs, siRNA, or inhibitors. The plates were then incubated for 3–7 days, then 100 μL of CellTiter-Glo reagent was added to lyse the cells. After a 10-min incubation at room temperature, luminescence was recorded in a luminometer with an integration time of 1 s per well.

For cell cycle analysis, transfected cells were harvested at 48 h and fixed at 4 °C. Propidium iodide (50 μg/mL) was used to stain the fixed cells. DNA content was analyzed using a FACSCalibur instrument (Becton Dickinson Bioscience). Cell cycle fractions were quantified with Multicycle Software (Phoenix Flow Systems) and analyzed by FlowJo software.

### Annexin V assay

Apoptosis was evaluated using a Muse Annexin V and Dead Cell kit (EMD Millipore). Cells were transfected with miRNAs or siRNAs or treated with inhibitors. After 72 h, cells were collected in 1% FBS medium, mixed with Muse Annexin V and Dead Cell Reagent, and analyzed using a Muse Cell Analyzer (EMD Millipore).

### Induction of adipogenic differentiation

As previously described^12^, confluent cells were cultured in differentiation-initiating medium (regular growth medium plus 100 nM insulin, 1 µM dexamethasone, 250 µM 3-isobutyl-1-methylxanthine (IBMX), 33 µM biotin, 17 µM pantothenic acid, and 5 µM of the PPARG agonist rosiglitazone). After 4 days, media were changed to maintenance medium (initiating medium lacking IBMX and rosiglitazone). Cells were fed with maintenance medium every 4 days thereafter.

### Oil red O staining

After 10 days of adipogenic differentiation, lipid droplets were stained with Oil Red O as described^12^. Stained cells were quantified, and three biological replicates (approximately 400–500 cells) were analyzed for each treatment group.

### RNA isolation and analysis

Total RNA was collected using TRIzol reagent (Invitrogen) and isolated by Direct-zol RNA mini prep kits (Zymo Research). cDNA was synthesized by the qScript DNA synthesis kit (Quanta Bioscience) or the QuantiMir RT kit (System Biosciences) according to the manufacturer’s instructions. TaqMan gene expression assays (Life Technology) were used for relative gene expression. miRNA expression levels were detected by using SYBR Green miRNA-specific primers. Quantitative real-time PCR (qRT-PCR) was performed on the ABI Prism 7900HT Sequence Detection System (Applied Biosystems). All transcript levels were normalized to levels of GAPDH transcript (for regular gene expression) or U6 snRNA (for miRNA expression). All primers are listed in Table S1.

### Immunoblotting

Cells were lysed in RIPA buffer (with 100x protease inhibitor cocktail and 25 μM MG132) and protein concentration was determined by the Lowry method. Equal amounts of total protein were isolated by SDS-PAGE and then transferred to PDVF membranes. After blocked, membranes were incubated with the appropriate primary antibodies, followed by HRP-conjugated secondary antibodies. Immunoreactive proteins were detected using Western Lightning chemiluminescence reagent.

### miRNA target prediction and network construction

We previously reported our methods of identifying 50 putative miR-193b targets^12^. Functional interactions of the 50 previously identified putative miR-193b targets were analyzed by STRING (https://string-db.org/). STRING interactions with a confidence score of 0.4 or higher are shown. Interactions were inferred from the STRING data mining and experimental databases.

### Statistical analysis

All data are presented as means ± S.E. Statistical significance was assessed using Student’s t test, and p values < 0.05 were considered statistically significant.

## Supplementary information

Supplementary data
