# Supplementary material for: miR-193b regulates tumorigenesis in liposarcoma cells via PDGFR, TGFβ, and Wnt signaling
Source: Sci Rep. 2019 Mar 1;9:3197. doi: 10.1038/s41598-019-39560-0 (PMC6397171; doi:10.1038/s41598-019-39560-0)
Supplement: Supplementary file 1 — Supplementary data [file 41598_2019_39560_MOESM1_ESM.pdf]

miR-193b regulates tumorigenesis in liposarcoma cells via PDGFR, TGFβ, and Wnt signaling

Ying Z. Mazzu, Yulan Hu, Yawei Shen, Thomas Tuschl, Samuel Singer

Supplementary data

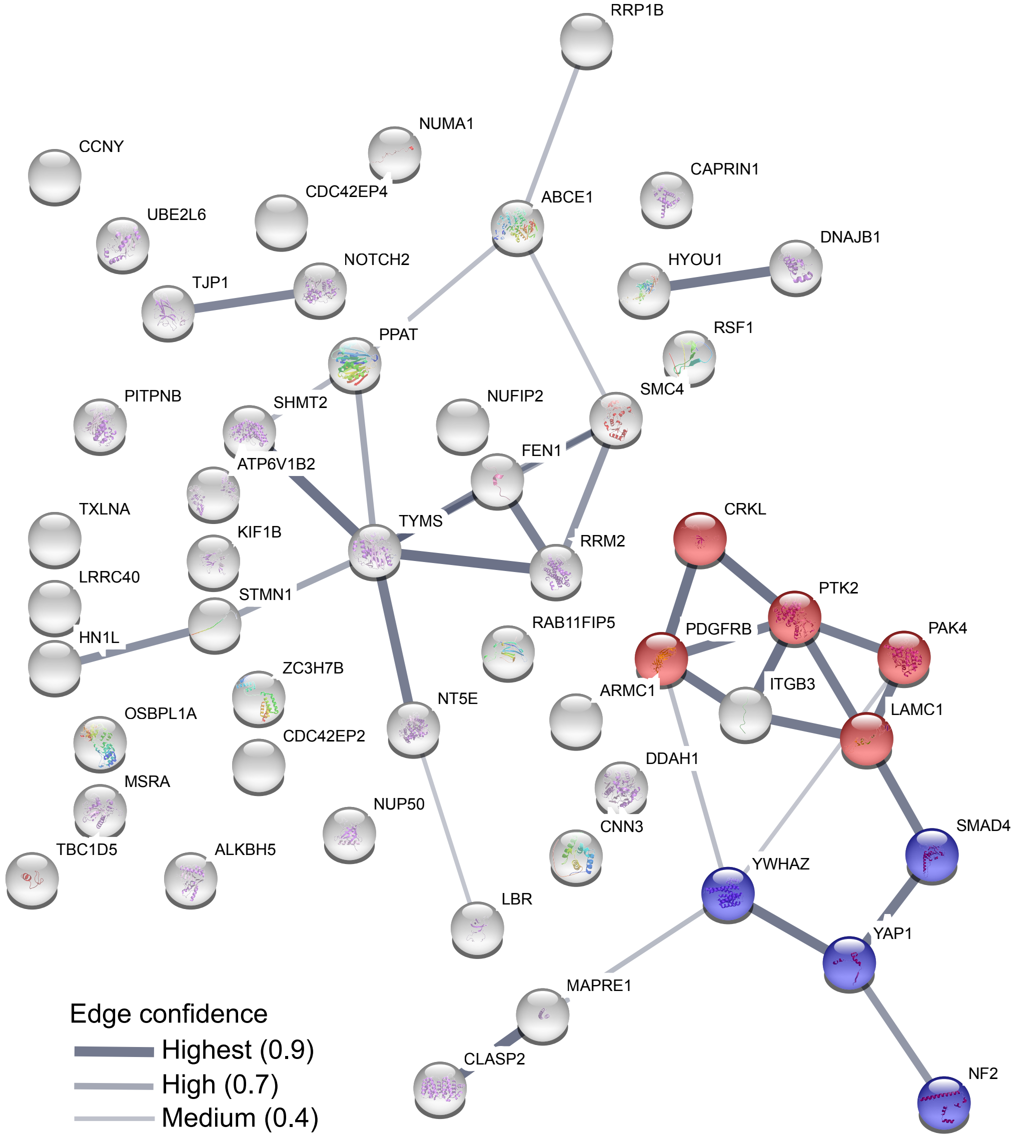

Pathway	Count in gene set	False discovery rate
Focal adhesion	5	0.0416
Hippo signaling	4	0.0459
MicroRNAs in cancer	4	0.0459

Figure S1. Protein-protein interaction network of putative miR-193b targets. STRING analysis was to construct interaction network among the 50 putative miR-193b targets identified previously [12]. STRING interactions with a confidence score of 0.4 or higher are shown and

highlighted in bold. Significantly interacting pathways are listed in the table; colors indicate related protein nodes.

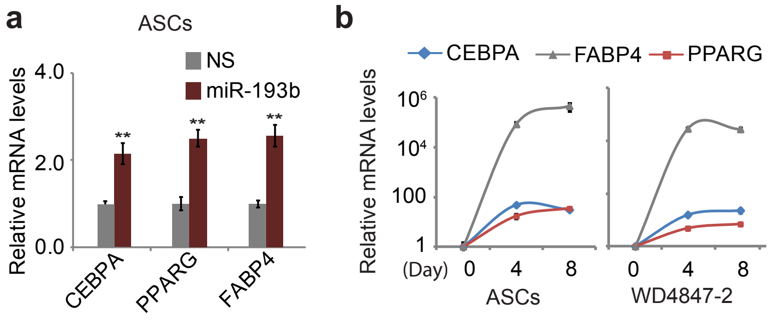

Figure S2. Adipogenic differentiation of ASCs and WD4847-2 cells. **a**, Effects of miR-193b on adipogenic differentiation marker mRNA levels during ASC differentiation. **b**, mRNA levels of differentiation markers during adipogenic differentiation in ASCs and WD4847-2 cells, as detected by qRT-PCR.

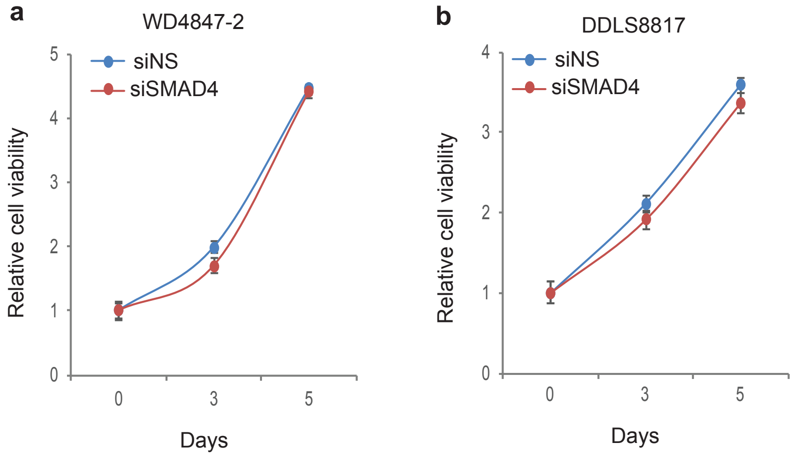

Figure S3. Knockdown of SMAD4 does not affect WDLS and DDLS cell proliferation. Cell proliferation was assessed at day 3 and day 5, after siRNA transfection in WD4847-2 (**a**) and DD8817 (**b**) cells. Values represent the mean ± SE of 3 independent experiments.

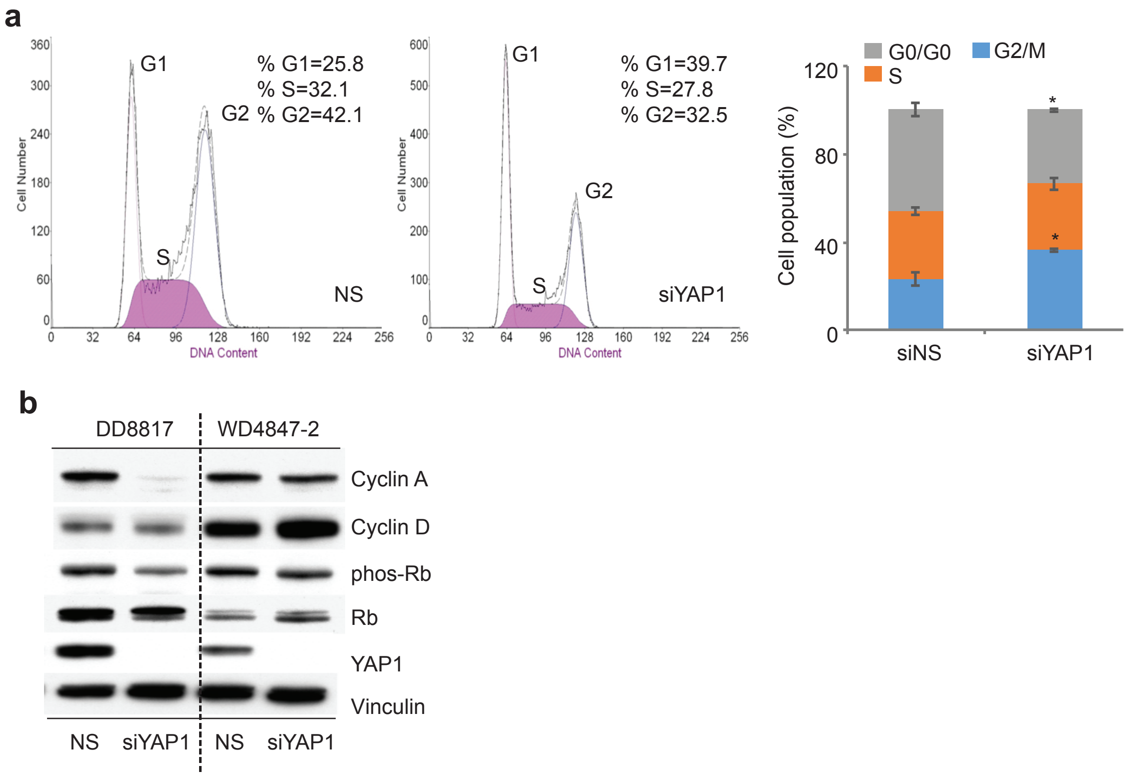

Figure S4. Knockdown of YAP1 by siRNA induces modest cell cycle arrest at G0/G1 in DDLS cells. a, Cell cycle FACS analysis was performed after 48h transfection with siRNAs. NS, nonspecific siRNA. Values represent the mean ± SE of 3 independent experiments. *, $p<0.05$. **b**, Protein expression of cell cycle markers detected by Western blotting in siRNA-treated DDLS cells (representative results from 2 independent experiments).

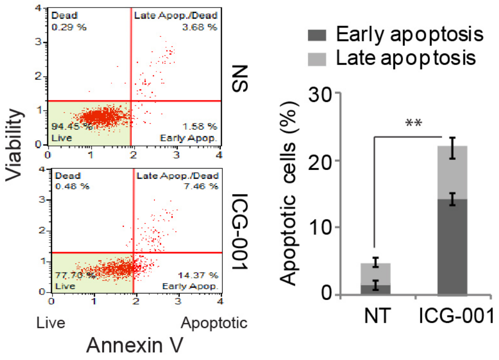

Figure S5. IC-001 induces apoptosis in DDLS cells. Apoptosis was detected in IC-001 (2.5 μM)-treated DD8817 cells by annexin V assay on day 2. Representative apoptosis profiling panels are shown. Values represent the mean ± SE of 3 independent experiments. **, $p < 0.01$ compared with vehicle or nonspecific miRNA or siRNA control.

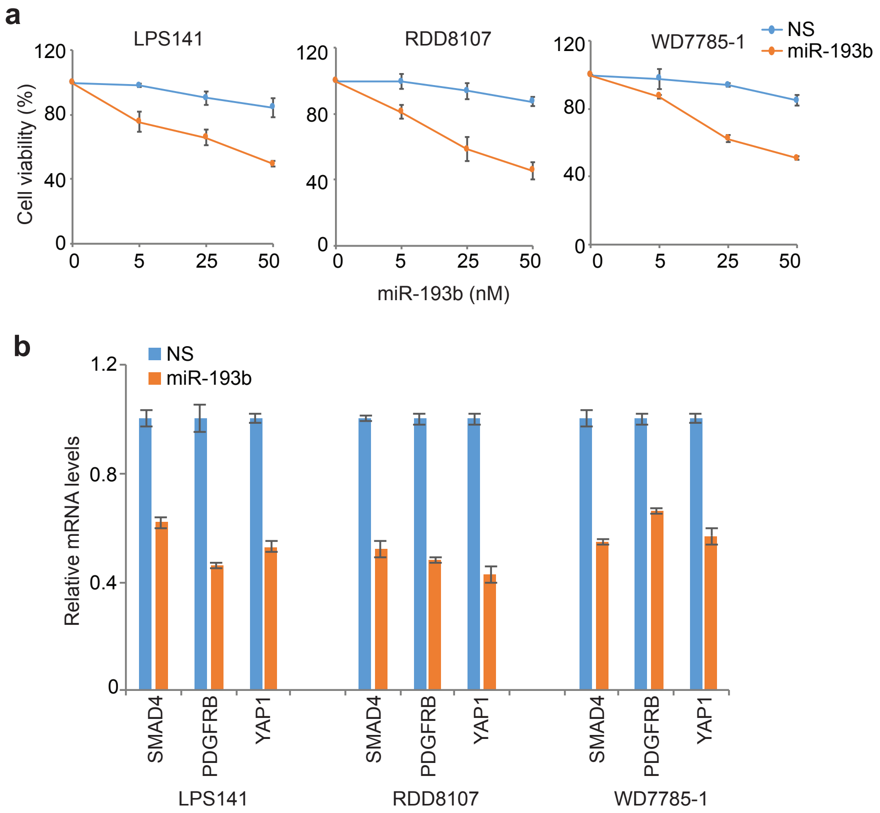

Figure S6. miR-193b slows growth of cells that express intermediate levels of miR-193b. The selected cell lines were previously shown to express miR-193b at levels between those of ASCs and the cell lines used in preceding experiments [12]. **a**, Proliferation of liposarcoma cells on day 5 after transfection with varying doses of miR-193b. **b**, Expression of miR-193b targets was evaluated by qRT-PCR after 3 days of transfection with miR-193b. Values represent the mean ± SE of 3 independent experiments.

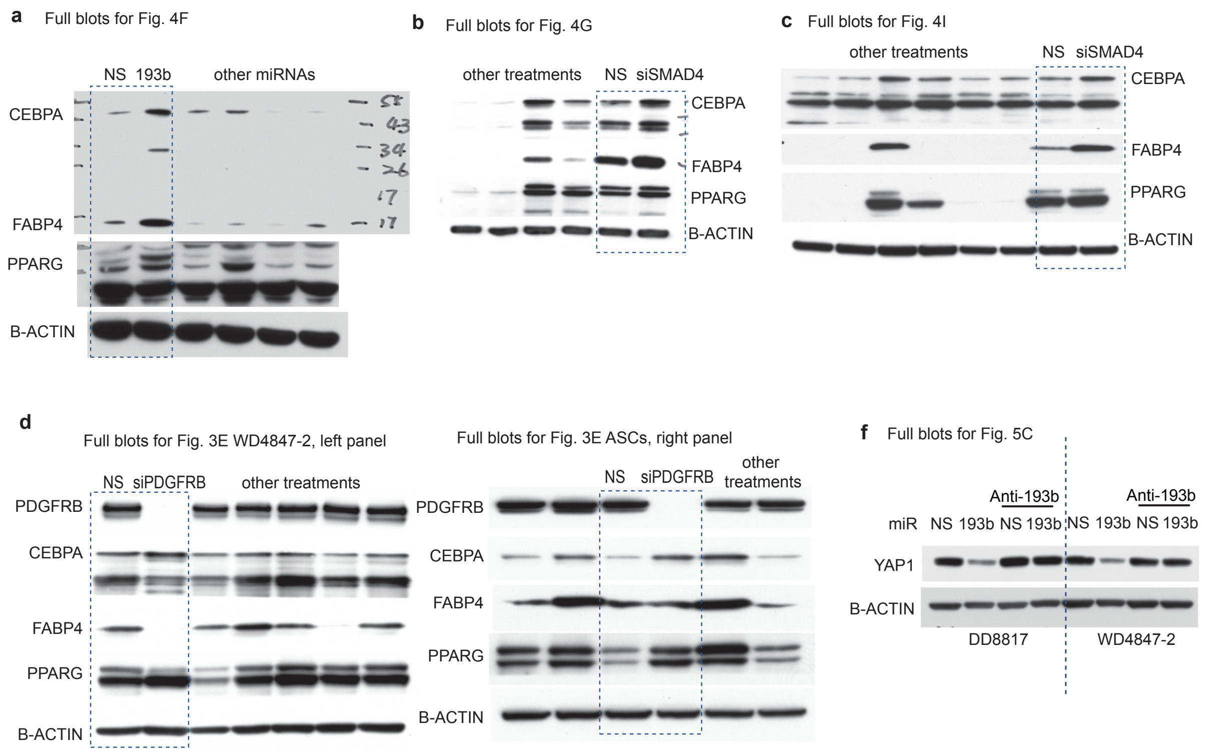

Figure S7. Full western blots for all figures.

Table S1. Oligonucleotide sequences for 3'UTR reporters, RT-PCR primers, and miRNAs.

Cloning primers for 3'UTR reporters

Reporter	Sequence
YAP1-WT	AAACTAGCGGCCGCAGCTACTTGGAAGGAGTGCCTATAATTTGCCAGTAGCCACAGAGCTACTTGGAAGGAGTGCCTATAATTTGCCAGTAGCCACAGT
YAP1-WT as	CTAGACTGTGGCTACTGGCAAATTATAGGCACTCCTTCCAAGTAGCTCTGTGGCTACTGGCAAATTATAGGCACTCCTTCCAAGTAGCTGCGGCCGCTAGTTT
YAP1-Mut	AAACTAGCGGCCGCAGCTACTTGGAAGGAGTGCCTATAATTTGCATATAGCCACAGAGCTACTTGGAAGGAGTGCCTATAATTTGCATATAGCCACAGT
YAP1-Mut as	CTAGACTGTGGCTATATGCAAATTATAGGCACTCCTTCCAAGTAGCTCTGTGGCTATATGCAAATTATAGGCACTCCTTCCAAGTAGCTGCGGCCGCTAGTTT
SMAD4-WT	AAACTAGCGGCCGCCCACTCGTAGCTTCTGCTTTGGGGACAACTGGTCAGTTGAAACCACTCGTAGCTTCTGCTTTGGGGACAACTGGTCAGTTGAAAT
SMAD4-WT as	CTAGATTTCAACTGACCAGTTGTCCCCAAAGCAGAAGCTACGAGTGGTTTCAACTGACCAGTTGTCCCCAAAGCAGAAGCTACGAGTGGGCGGCCGCTAGTTT
SMAD4-Mut	AAACTAGCGGCCGCCCACTCGTAGCTTCTGCTTTCCTAACAACTCATGCATTGAAACCACTCGTAGCTTCTGCTTTCCTAACAACTCATGCATTGAAAT
SMAD4-Mut as	CTAGATTTCAATGCATGAGTTGTTAGGAAAGCAGAAGCTACGAGTGGTTTCAATGCATGAGTTGTTAGGAAAGCAGAAGCTACGAGTGGGCGGCCGCTAGTTT
PDGFRB-WT	AAACTAGCGGCCGCATGTGCCAGTGTGGAGTGGCCACGTGTGTGTGCCAGTATATGATGTGCCAGTGTGGAGTGGCCACGTGTGTGTGCCAGTATATGT
PDGFRB-WT-as	CTAGCTAGACATATACTGGCACACACACGTGGCCACTCCACACTGGCACATCATATACTGGCACACACACGTGGCCACTCCACACTGGCACATGCGGCCGCTAGTTT
PDGFRB-WT	AAACTAGCGGCCGCATGTGCCAGTGTGGAGTGGCCACGTGTGTGTCATGGCATATGATGTGCCAGTGTGGAGTGGCCACGTGTGTGTCATGGCATATGT
PDGFRB-WT-as	CTAGACATATGCCATGACACACACGTGGCCACTCCACACTGGCACATCATATGCCATGACACACACGTGGCCACTCCACACTGGCACATGCGGCCGCTAGTTT

Taqman primers

Primer	Assay ID (from Thermo Fisher Scientific)
SMAD4	Hs00929647_m1
PDGFRB	Hs01019589_m1
YAP1	Hs00902712_g1
BIRC5	Hs04194392_s1
PTTG	Hs00747713_sH
CEBPA	Hs00269972_s1
PPARG	Hs00234592_m1
FABP4	Hs00609791_m1
GAPDH	Hs99999905_m1

miRNA primers:

Primer	Sequence
miR-193b-3p	AACTGGCCCTCAAAGTCCCGCT
